# Supplementary material for: Suppressed excitonic effects enable high mobility and high-yield photoconductivity in a pyridine-coordinated two-dimensional polymer crystal
Source: Nat Commun. 2026 Aug 21;17:8815. doi: 10.1038/s41467-026-77017-x (PMC13498572; doi:10.1038/s41467-026-77017-x)
Supplement: Supplementary file 1 [file 41467_2026_77017_MOESM1_ESM.pdf]

Supplementary Information for

Suppressed excitonic effects enable high mobility and high-yield photoconductivity in a pyridine-coordinated two-dimensional polymer crystal

Shuai Fu[1,2,#,*], Ye Yang[1,3,#], Guoquan Gao[4,#], Shuangjie Zhao[1,#], Miroslav Položij[1,5,6], Tong Zhu[4], Lei Gao[2], Thomas Heine[1,5,6,7], Zhiyong Wang[1,3]*, Mischa Bonn[2]*, Xinliang Feng[1,3]*

[1]Center for Advancing Electronics Dresden and Faculty of Chemistry and Food Chemistry, Technische Universität Dresden, Dresden, Germany

[2]Max Planck Institute for Polymer Research, Mainz, Germany

[3]Max Planck Institute of Microstructure Physics, Halle (Saale), Germany

[4]Laser Micro/Nano Fabrication Laboratory, School of Mechanical Engineering, Beijing Institute of Technology, Beijing, China

[5]Helmholtz-Zentrum Dresden-Rossendorf, HZDR, Dresden, Germany

[6]Center for Advanced Systems Understanding, CASUS, Görlitz, Germany

[7]Department of Chemistry and IBS Center for Nanomedicine, Yonsei University, Seoul, Republic of Korea

[#]These authors contributed equally: Shuai Fu, Ye Yang, Guoquan Gao, Shuangjie Zhao

*E-Mail: shuai.fu@tu-dresden.de; zhiyong.wang@mpi-halle.mpg.de; bonn@mpip-mainz.mpg.de; xinliang.feng@mpi-halle.mpg.de

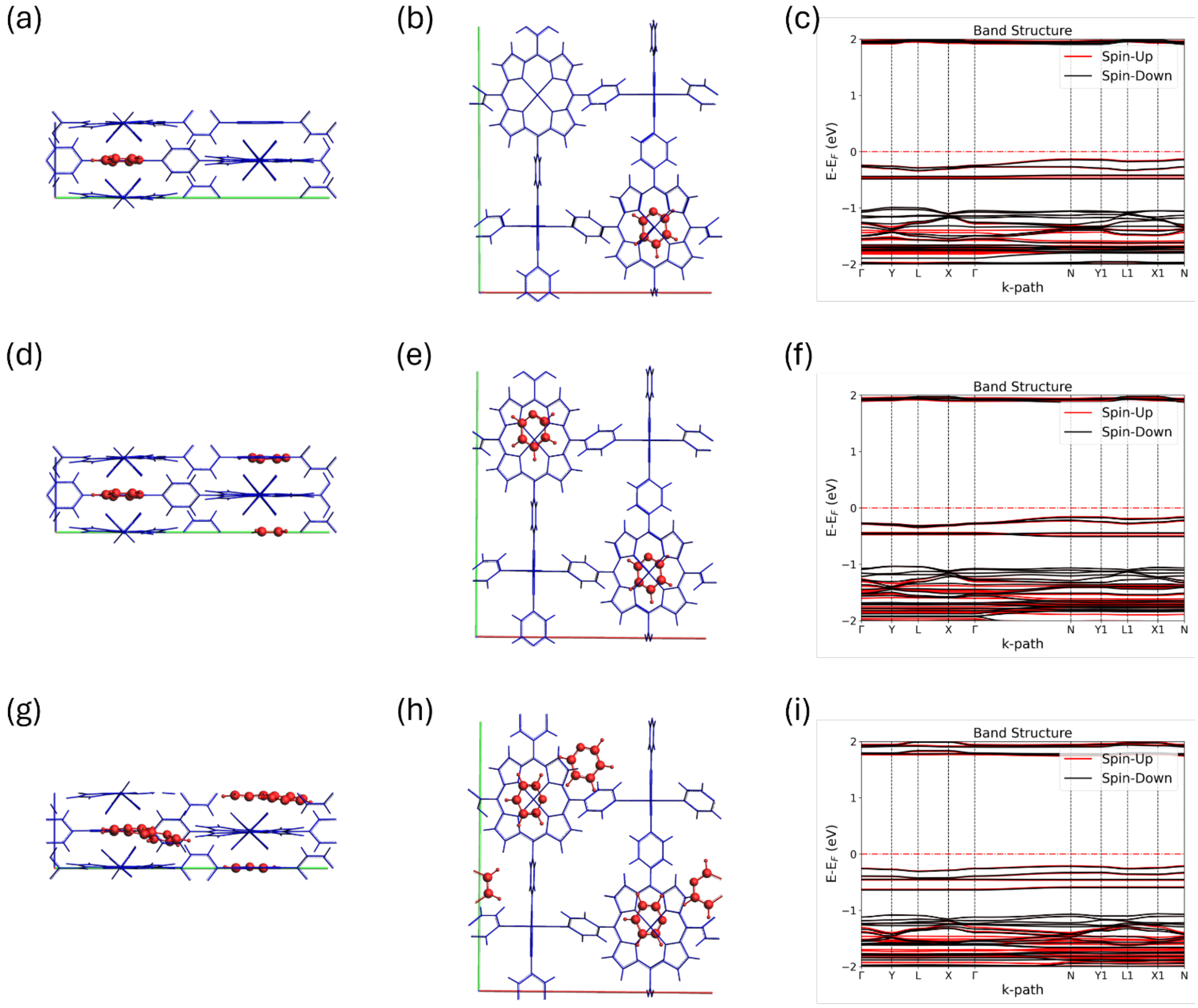

Supplementary Fig. 1 Structures and electrical band structures of PI-DY2DP with pyridine adsorbed at the preferential adsorption sites between AB-stacked layers. Top row: Structure (a,b) and electrical band structure (c) of PI-DY2DP with one pyridine molecule per unit cell; Middle row: Structure (d,e) and electrical band structure (f) of PI-DY2DP with two pyridine molecules per unit cell (one per Cu center); Bottom row: Structure (g,h) and electrical band structure (i) of PI-DY2DP with four pyridine molecules per unit cell (two per Cu center).

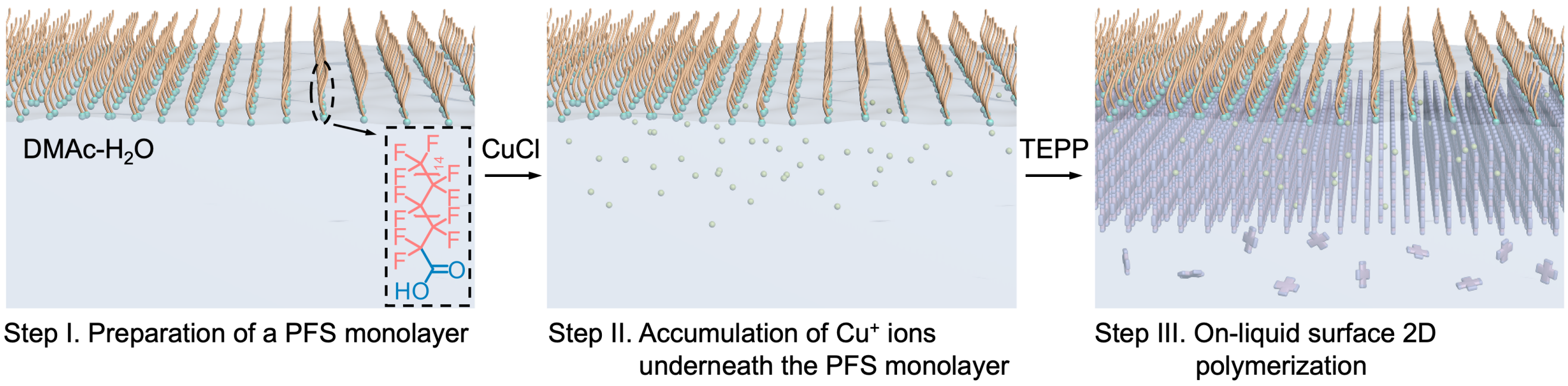

Supplementary Fig. 2 Schematic of on-organic-liquid surfactant-monolayer-assisted interfacial synthesis (O-SMAIS) strategy. In step I, a perfluoro-surfactant (PFS) monolayer was formed on the N,N-dimethylacetamide/water (DMAc-H_2O) surface, followed by the introduction of an aqueous CuCl solution, with or without pyridine, as a catalyst for Glaser coupling. In step II, electrostatic interactions led to the enrichment of Cu^+ ions beneath the anionic PFS monolayer, creating a catalyst-rich interfacial region. 5,10,15,20-tetrakis(4-ethynylphenyl)porphyrin (TEPP) monomers were then injected into the subphase, where coordination between terminal alkynes and Cu^+ induced vertical assembly and triggered interfacial polymerization. In step III, after 24 h, the resulting 2DPs were transferred onto solid substrates by horizontal lifting for characterizations.

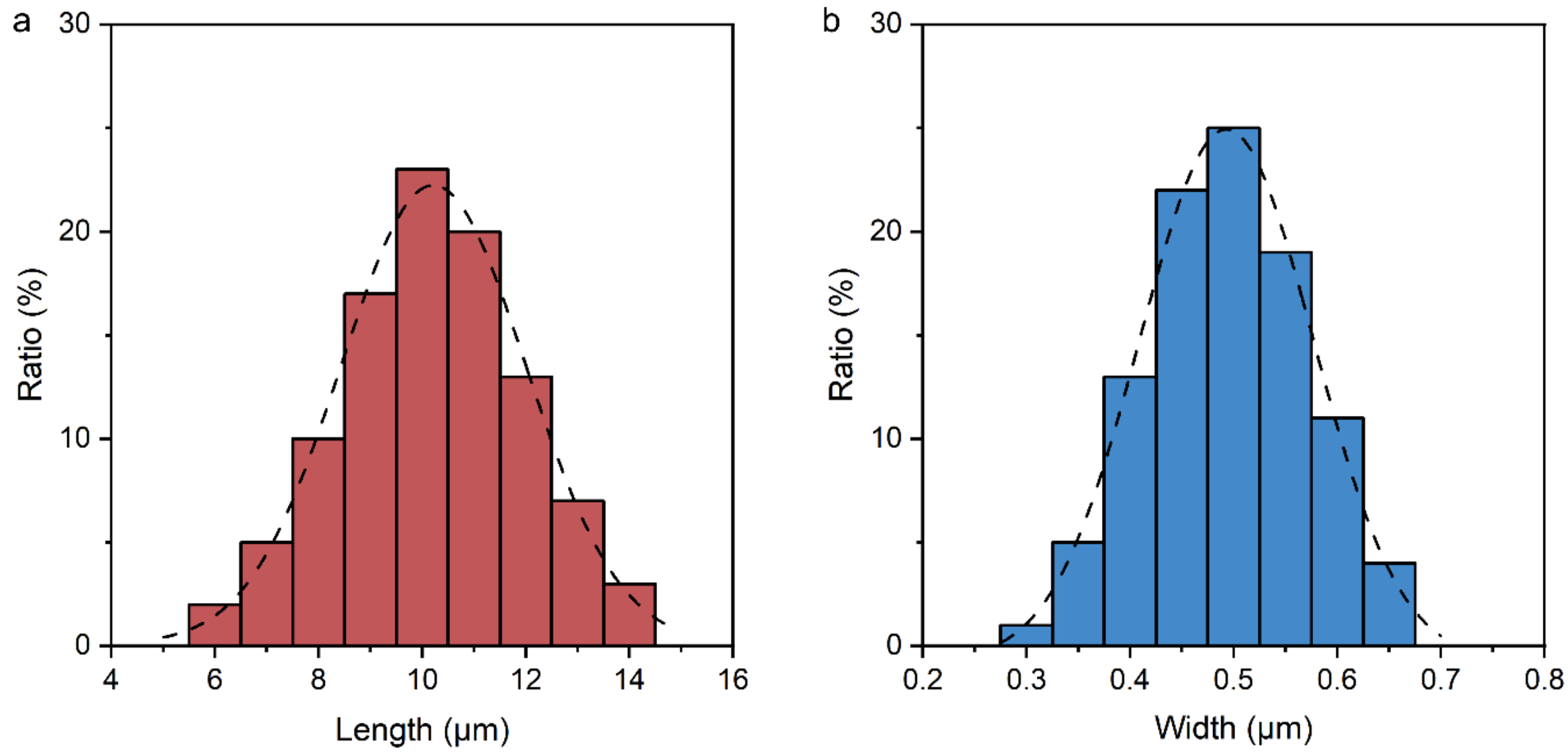

Supplementary Fig. 3 Statistical analysis of PI-DY2DP crystal dimensions. **a,** Length distribution. **b,** Width distribution.

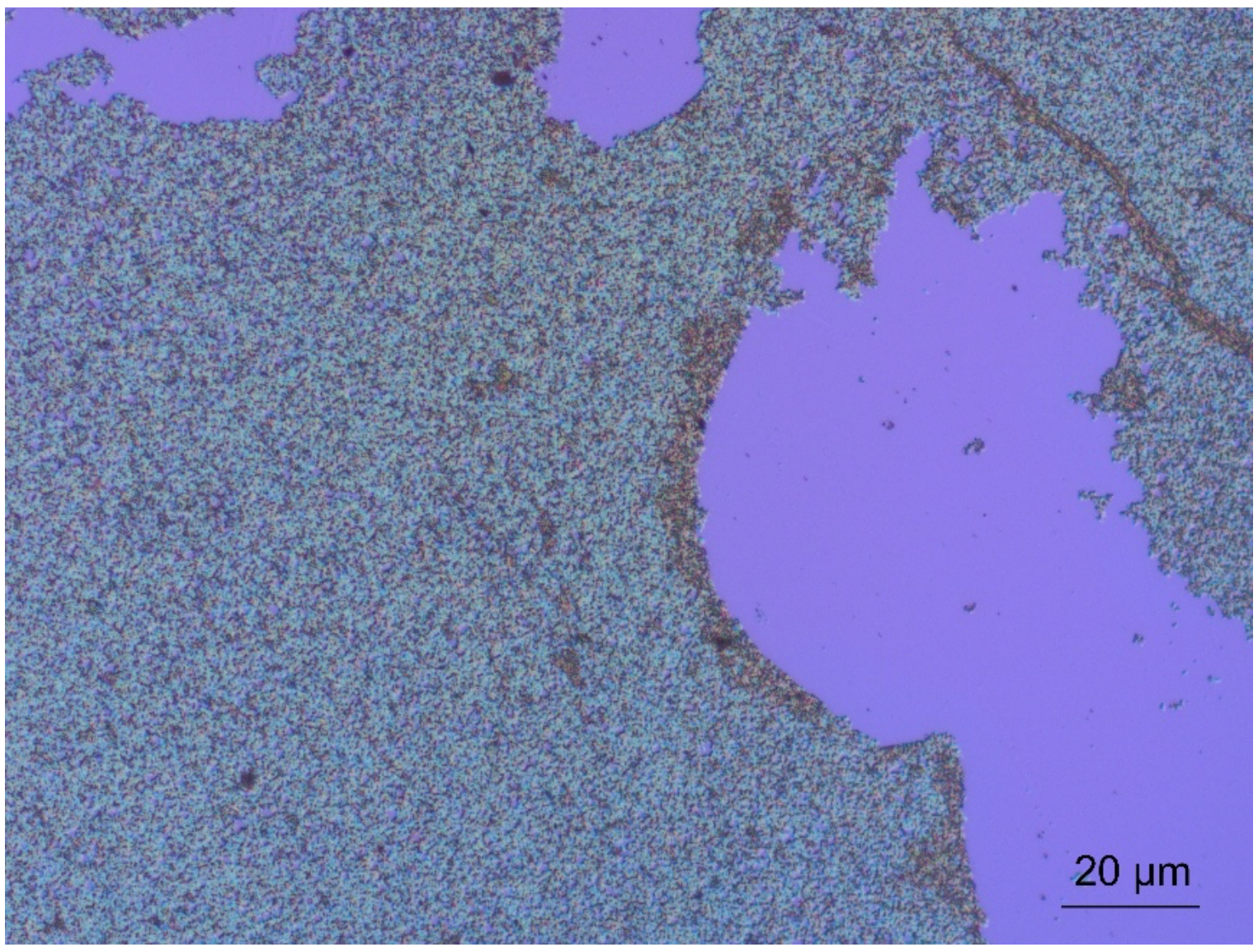

Supplementary Fig. 4 Optical microscopy (OM) images of PF-DY2DP. The resulting PF-DY2DP is in the form of nanocrystals with typical lateral dimensions of ~0.3 × 0.3 μm^2.

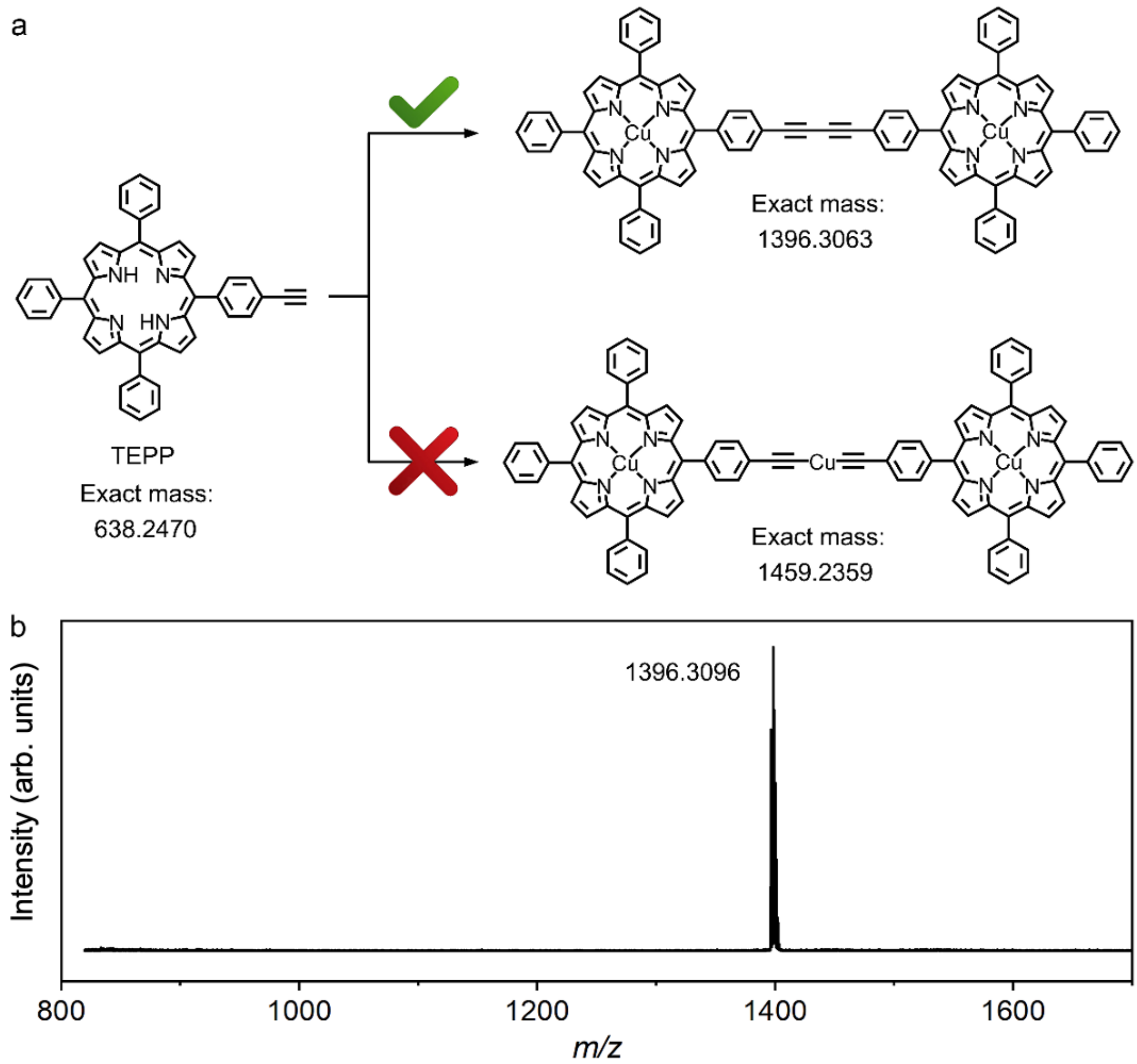

Supplementary Fig. 5 Model reaction and products. (a) Schematic of model reactions. (b) MALDI-TOF MS analysis of the model products on the N,N-dimethylacetamide/water (DMAc-H_2O) surface.

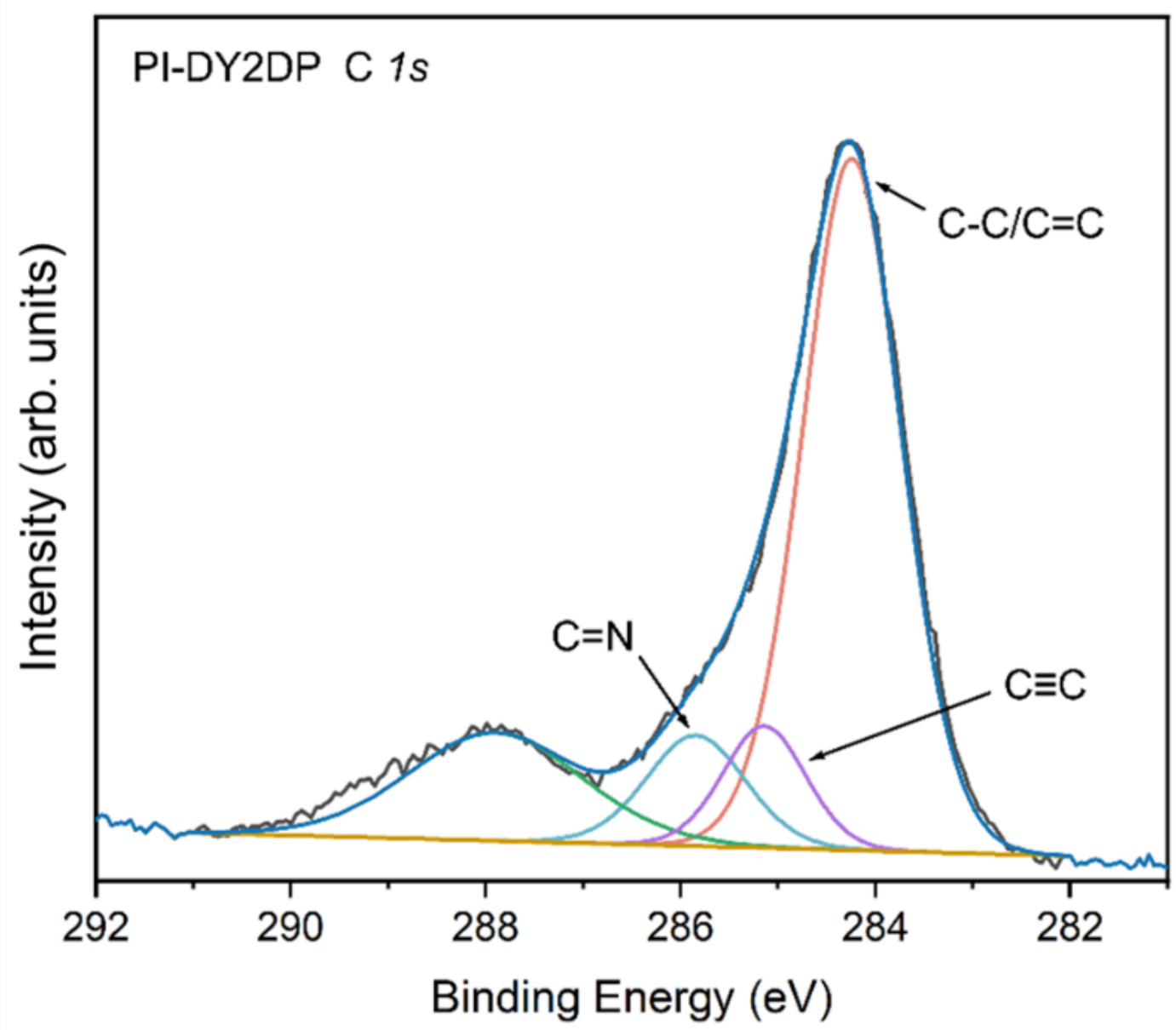

Supplementary Fig. 6 High-resolution XPS C *1s* spectrum of PI-DY2DP. Signals were observed at ~284.3 eV, ~285.1 eV, and ~285.8 eV, which were attributed to C–C/C=C, *sp*-hybridized carbon atoms associated with C≡C bonds, and C=N components, respectively.

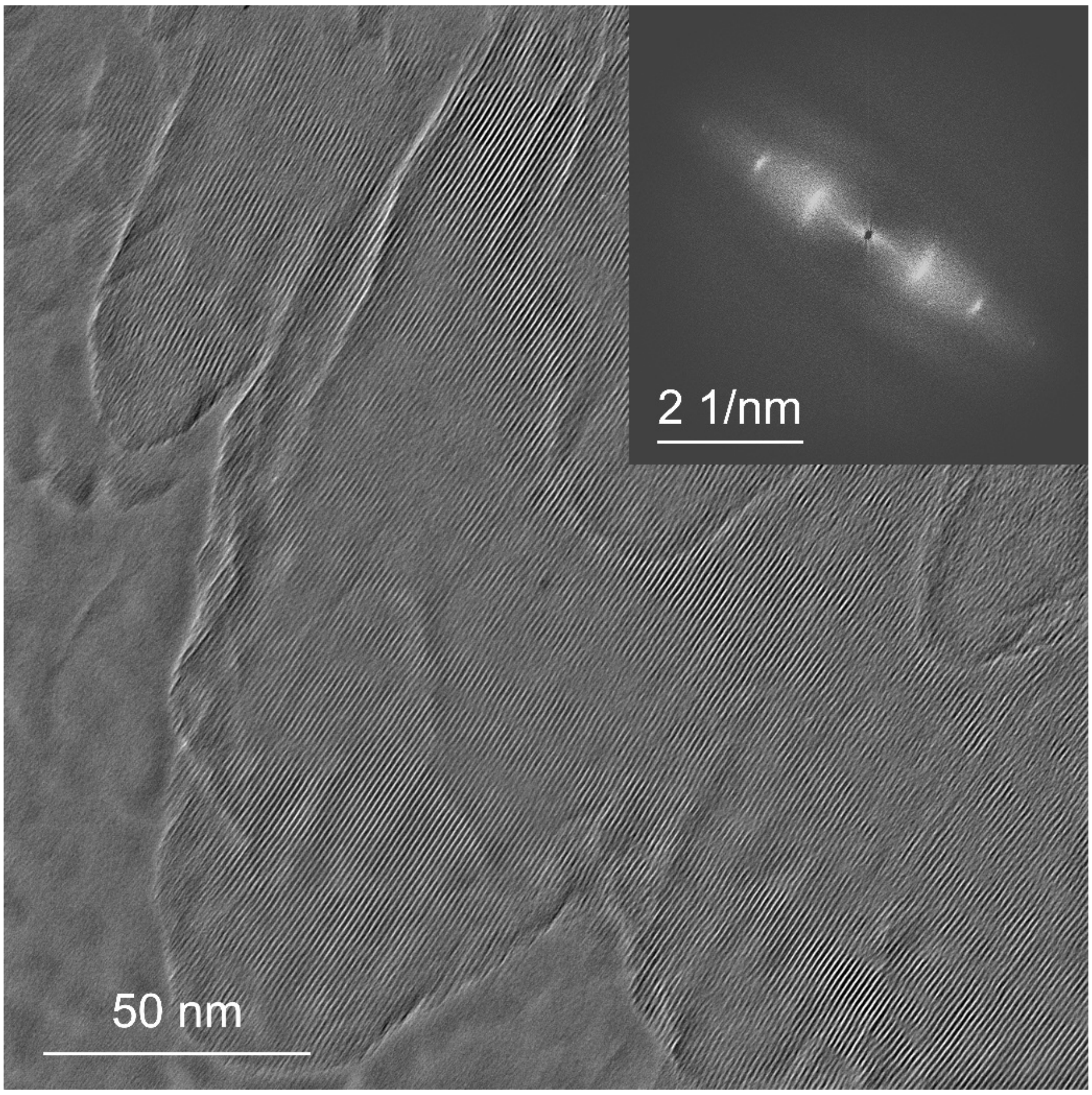

Supplementary Fig. 7 TEM image and the FFT pattern of PF-DY2DP. The *d*-spacing of the lattice fringes is approximately 1.1 nm.

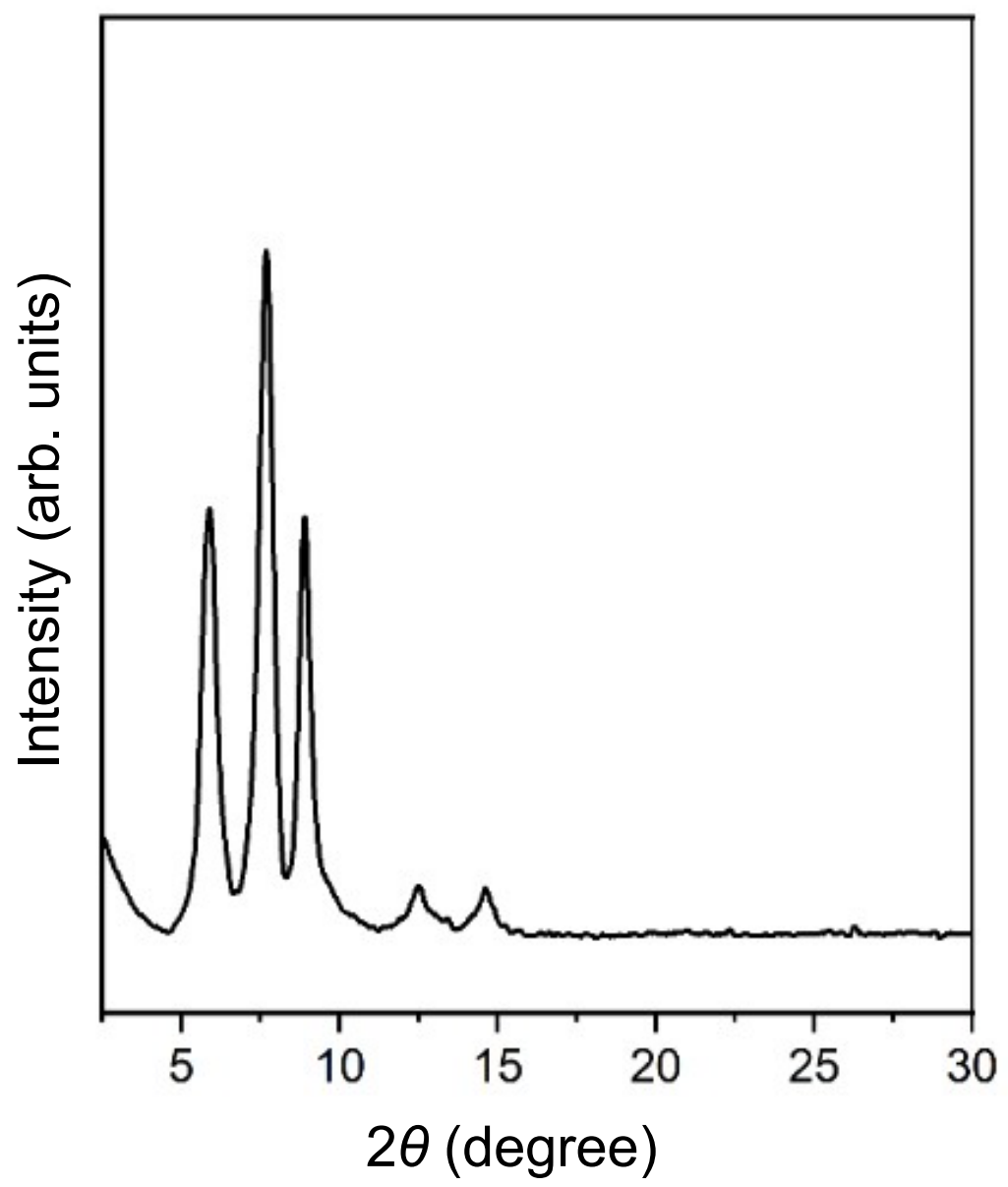

Supplementary Fig. 8 XRD patterns of PF-DY2DP. Diffraction features were observed at 2θ = 5.89°, 7.69°, 8.89°, 12.51°, and 14.62°.

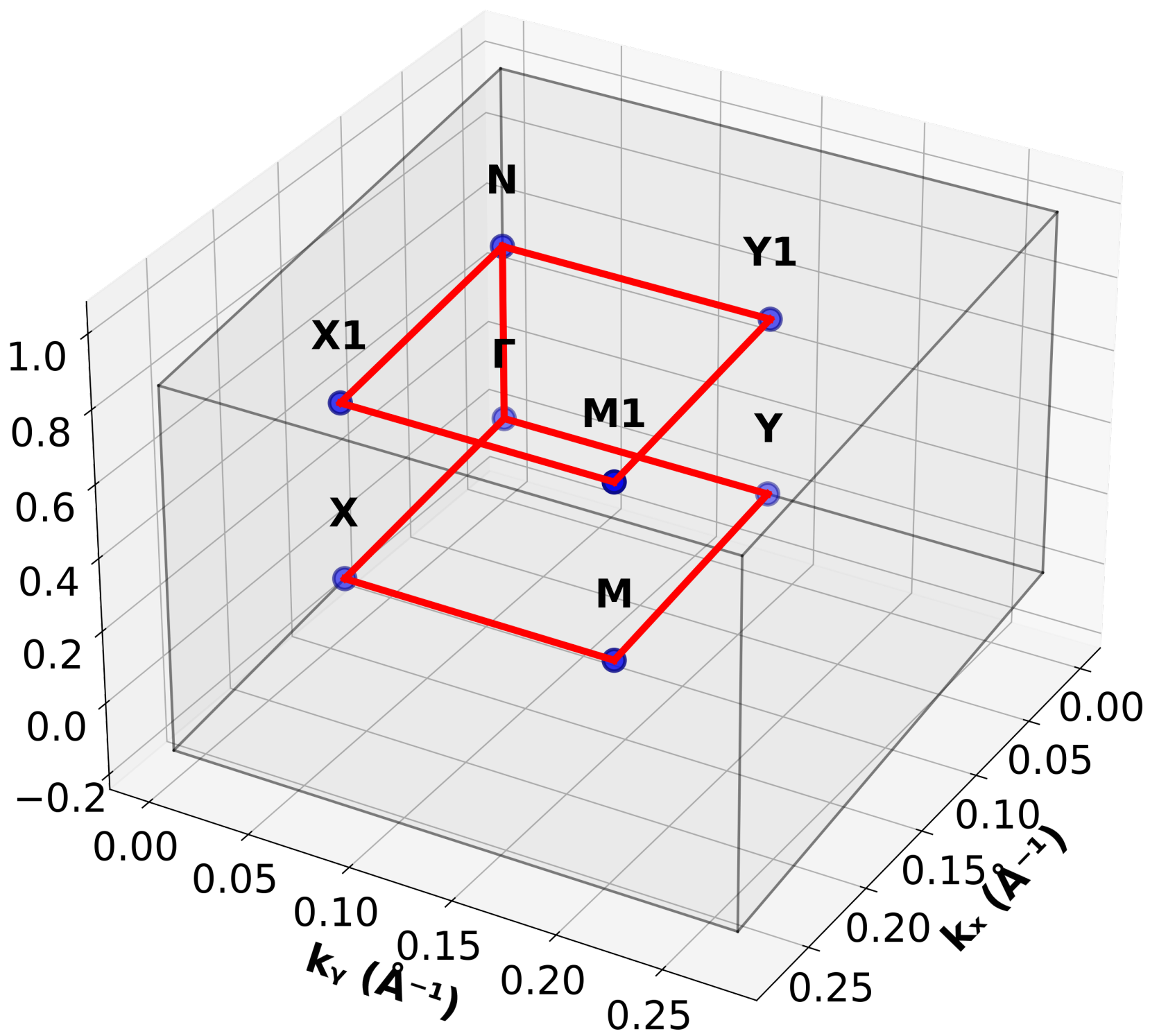

Supplementary Fig. 9 First Brillouin zone of DY2DP. The high-symmetry *k* points and the corresponding reciprocal-space path used for the electronic band-structure calculations are highlighted.

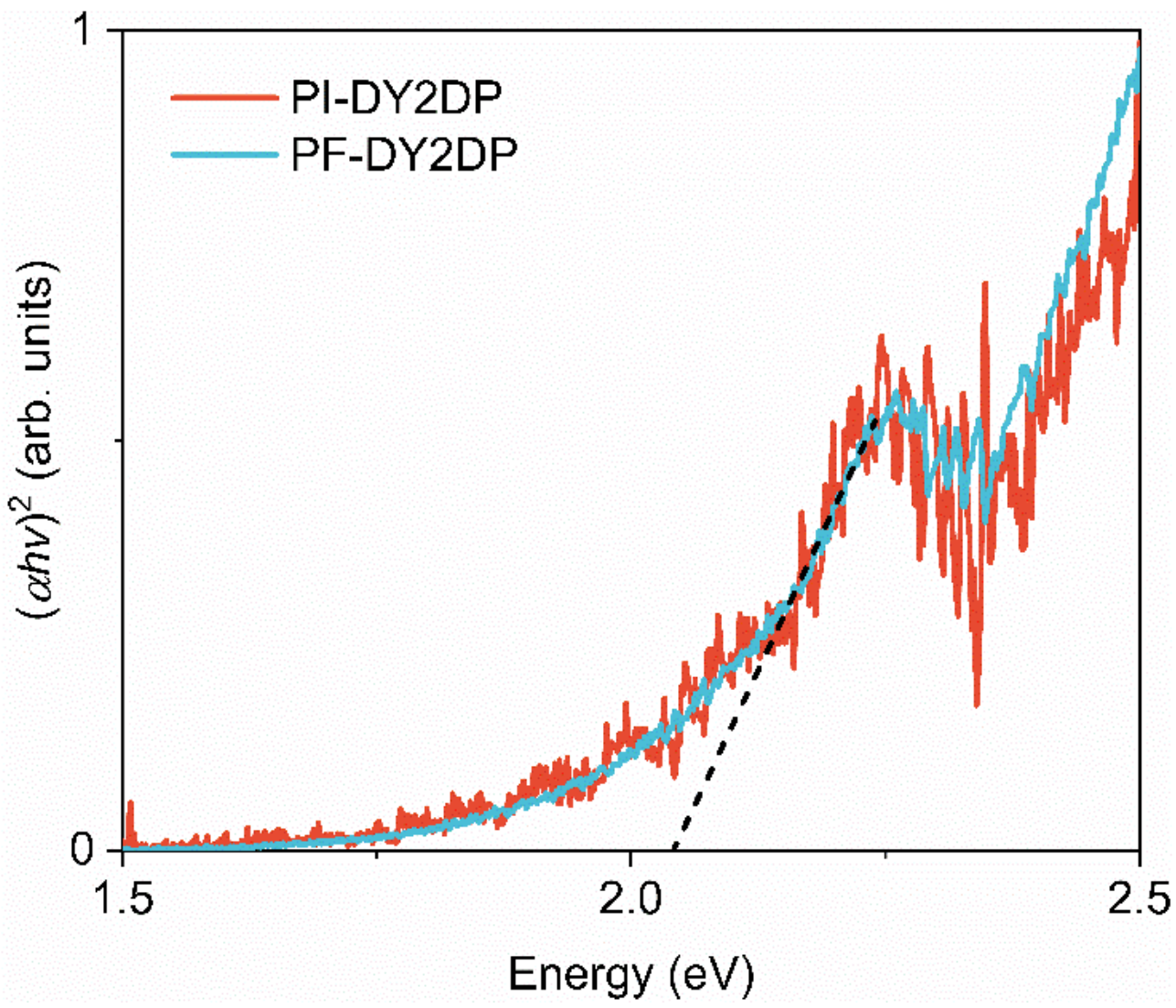

Supplementary Fig. 10 Optical absorption spectra of PI-DY2DP and PF-DY2DP plotted in Tauc representation. The dashed line denotes the extracted optical bandgap of 2.04 eV.

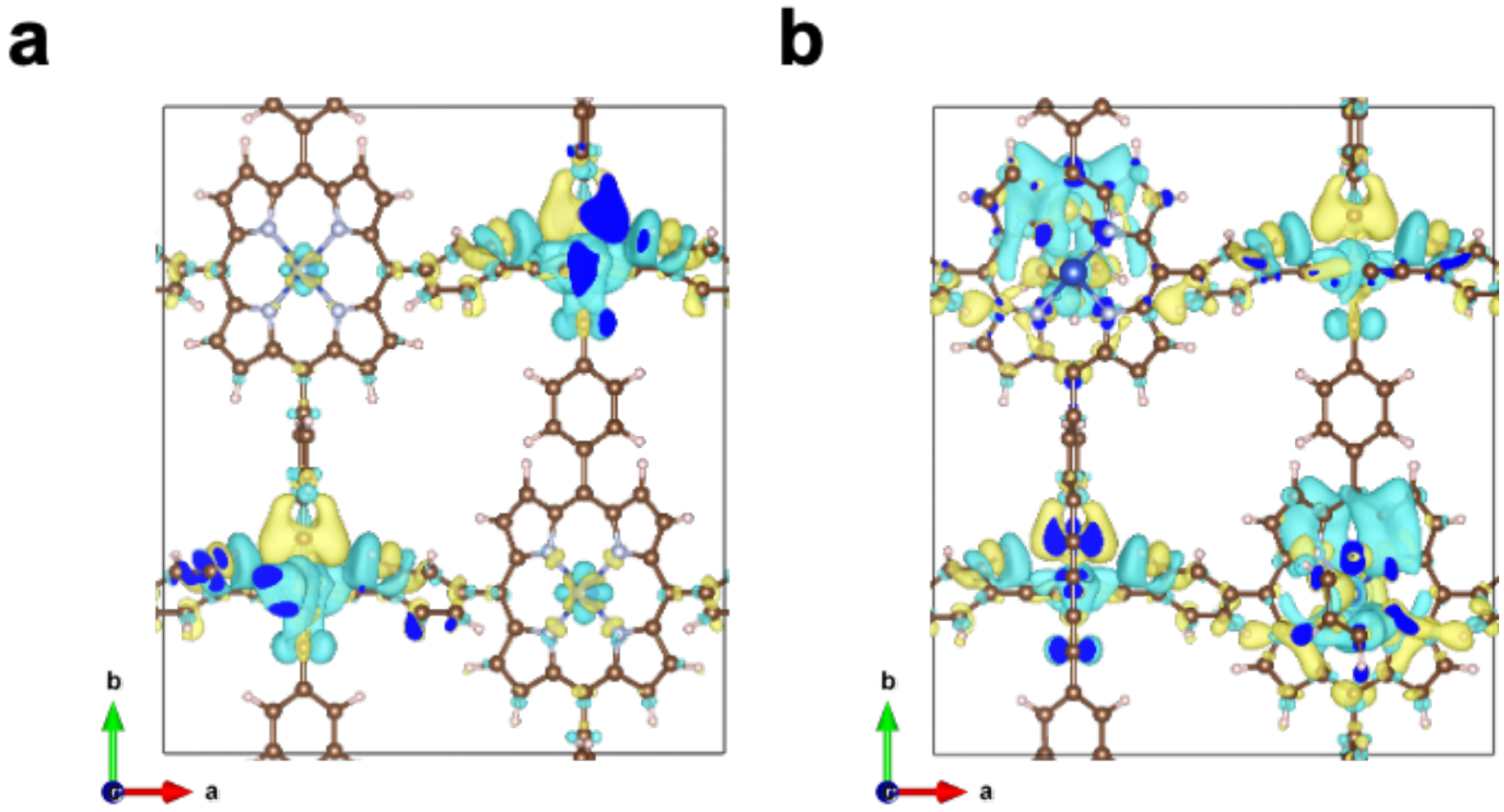

Supplementary Fig. 11 Charge-density-difference (CDD) maps. Top view of CDD maps of PF-DY2DP (a) and PI-DY2DP (b).

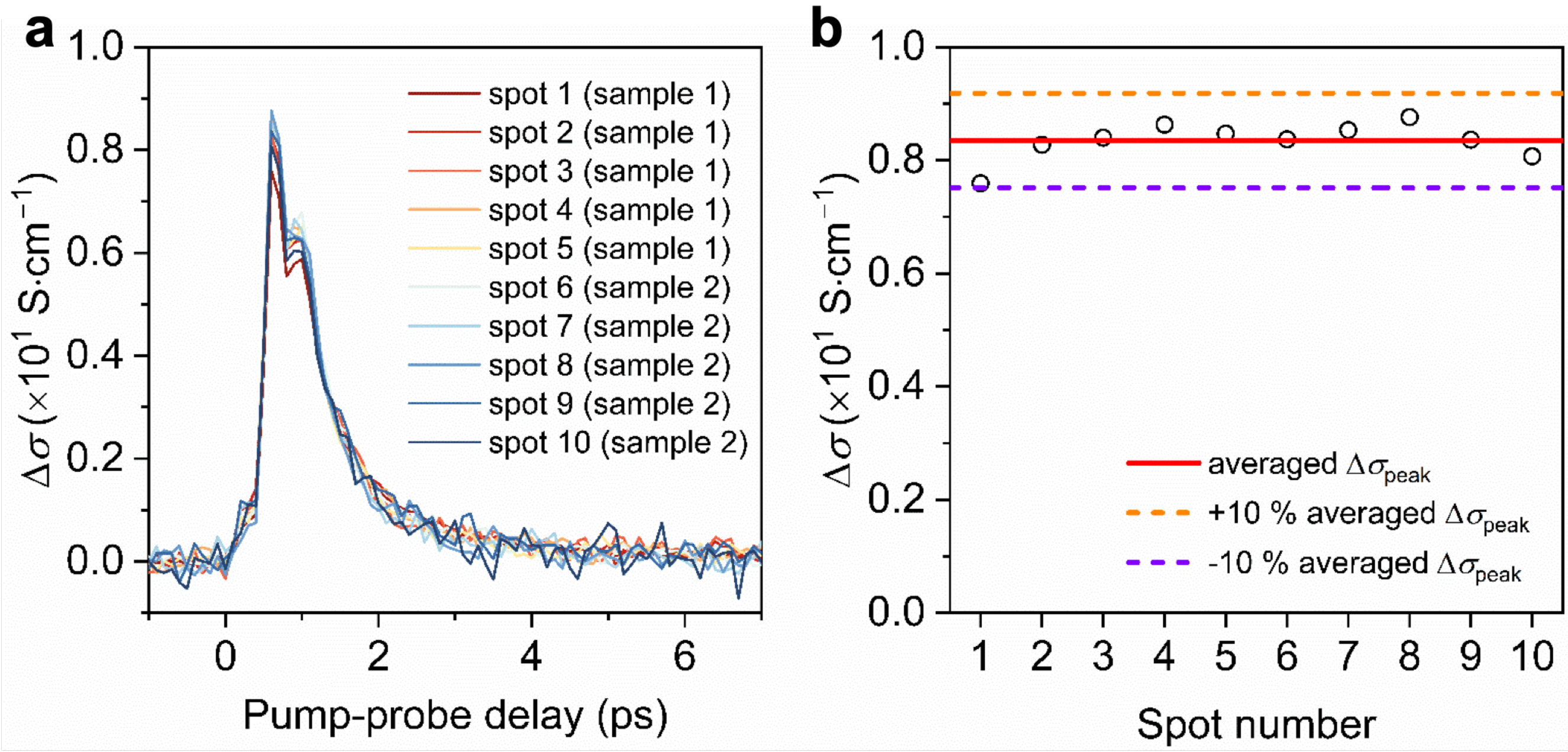

Supplementary Fig. 12 Reproducibility of the THz photoconductivity response of PI-DY2DP. (a) Time-resolved THz photoconductivity ($\Delta\sigma$) of PI-DY2DP measured at $N_{abs} = 1.19 \times 10^{12}$ cm^{-2}, reproduced across ten spatially separated positions and two independently synthesized batches. (b) Statistical distribution of the peak photoconductivity ($\Delta\sigma_{peak}$) extracted from measurements at different sample spots, showing variations within ~10%.

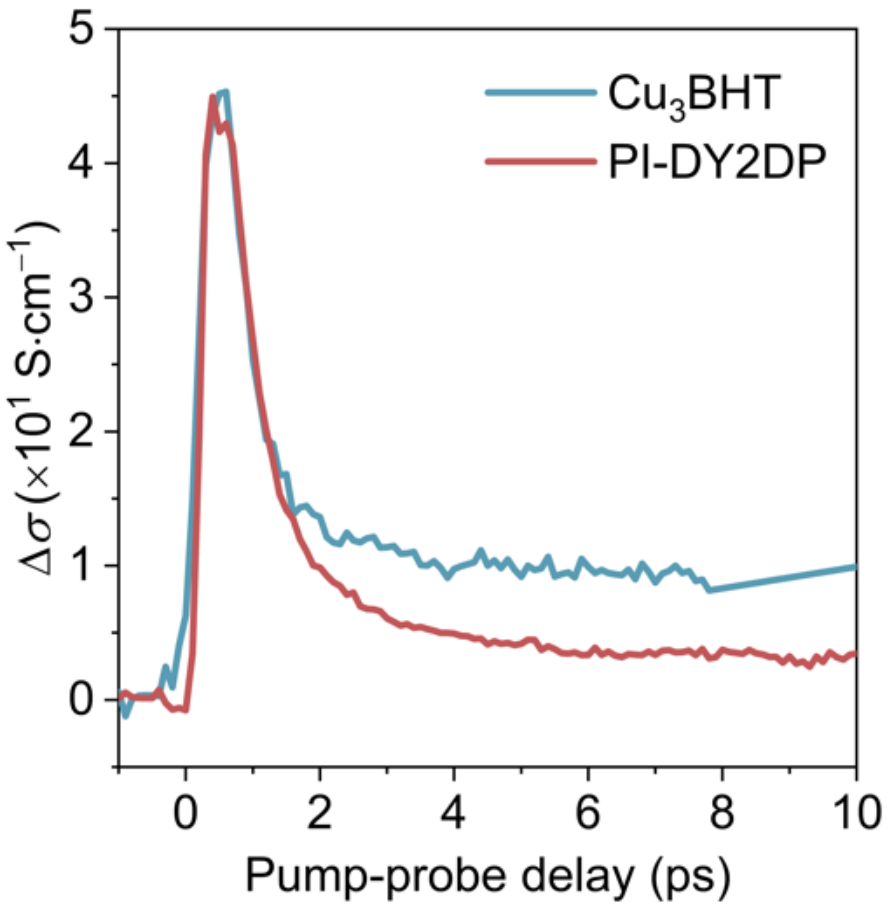

Supplementary Fig. 13 Comparison of time-resolved $\Delta\sigma$ of Cu_3BHT (BHT = benzenehexathiol) (3.10 eV photoexcitation at N_{abs} = in 9.60×10^{14} cm^{-2}) and PI-DY2DP (3.10 eV photoexcitation at $N_{\mathrm{abs}} = 7.00 \times 10^{12}$ cm^{-2}, same as in Fig. 2g) at room temperature. Data for Cu_3BHT are reproduced from reference[1].

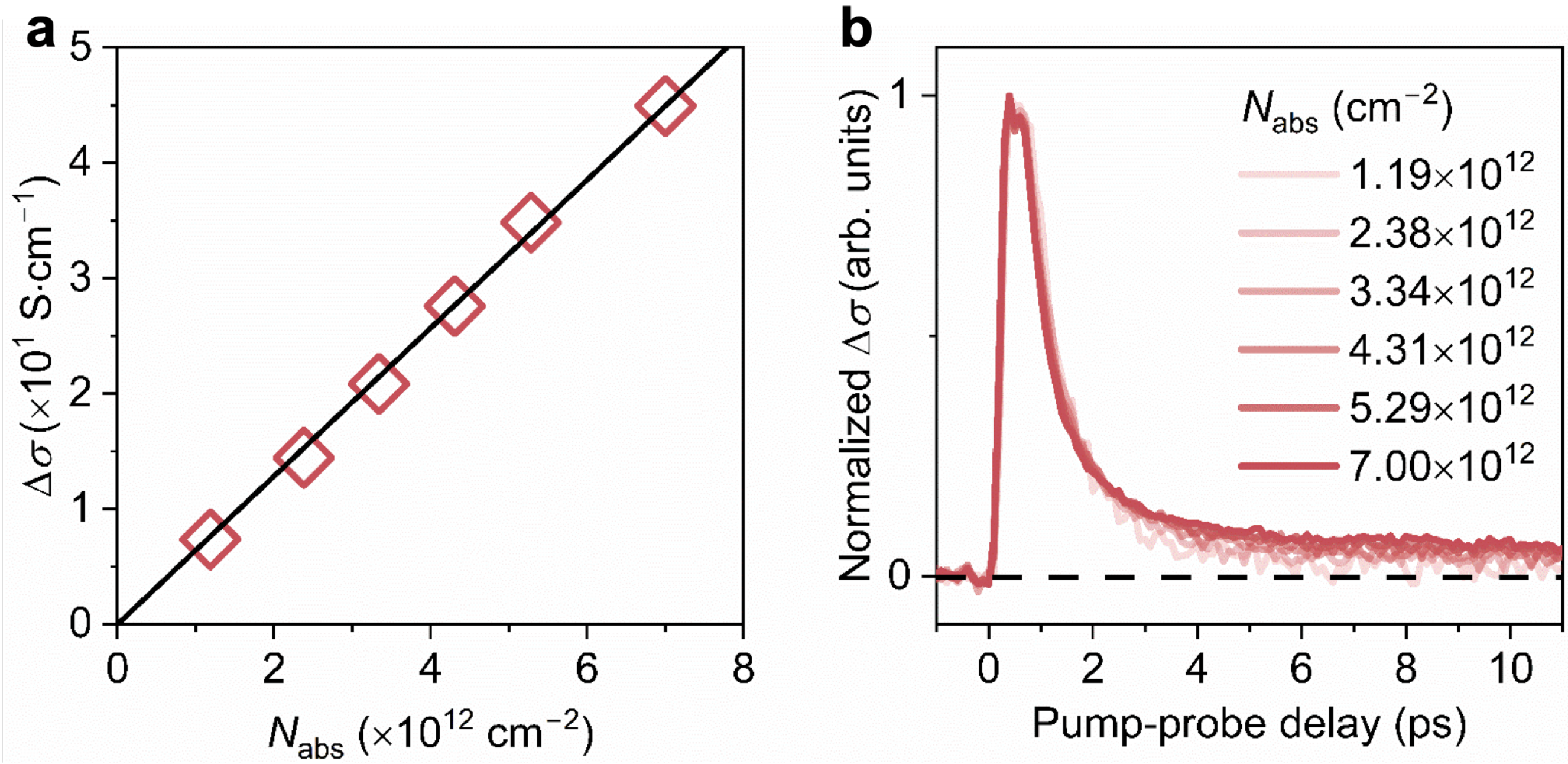

Supplementary Fig. 14 Pump fluence-dependent time-resolved $\Delta\sigma$ of PI-DY2DP. (a) $\Delta\sigma_{\mathrm{peak}}$ as a function of N_{abs}. The solid line represents a linear fit. (b) Normalized dynamics measured at N_{abs} ranging from 1.19×10^{12} to 7.00×10^{12} cm^{-2}.

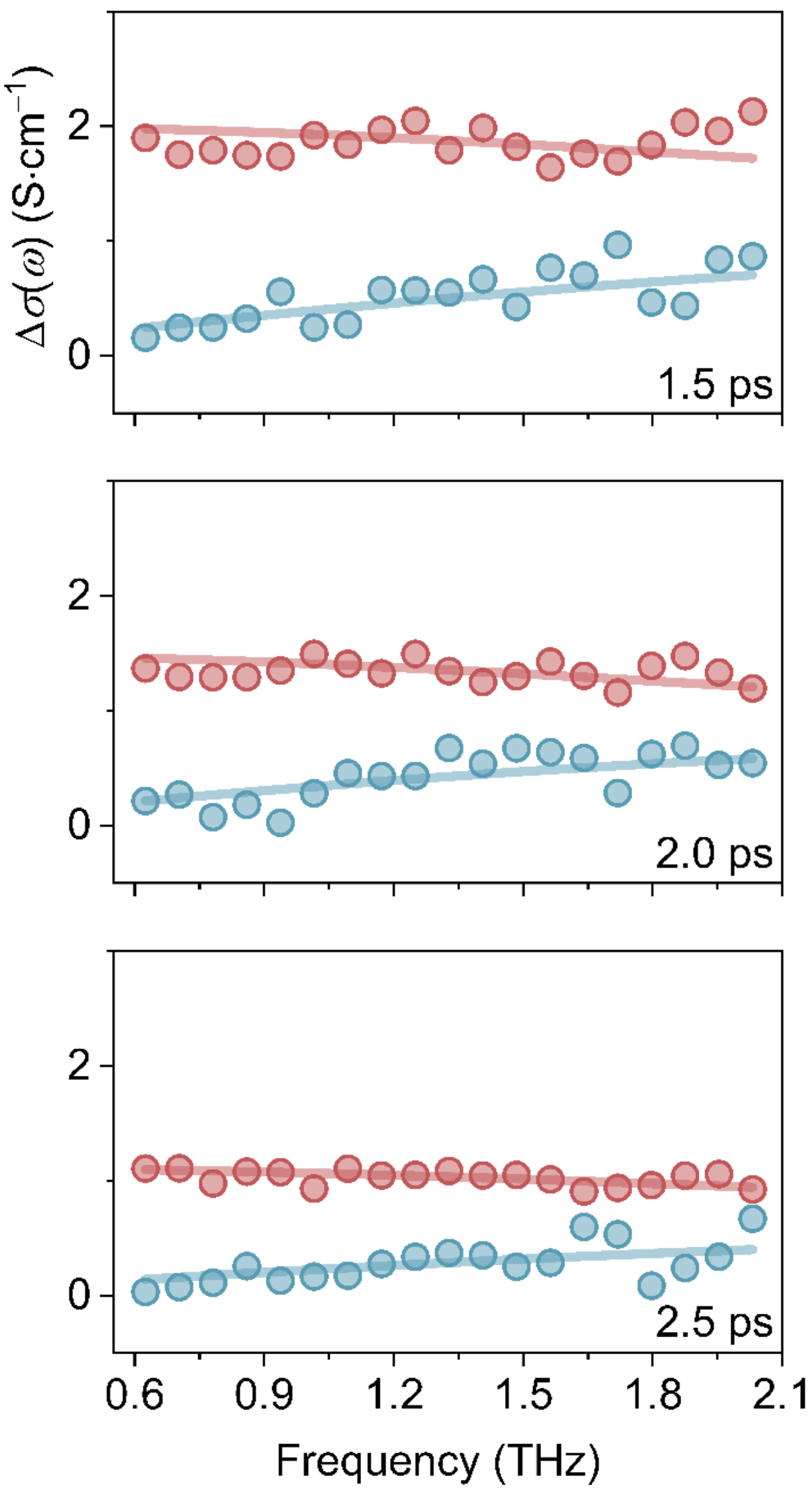

Supplementary Fig. 15 Frequency-resolved complex THz photoconductivity, $\Delta\sigma(\omega)$, measured at 1.5 ps (top), 2 ps (middle), and 2.5 ps (bottom) after σ_{peak} for PI-DY2DP at room temperature. Red and blue symbols denote the real and imaginary components of $\Delta\sigma(\omega)$, respectively, and solid lines represent the corresponding Drude fits.

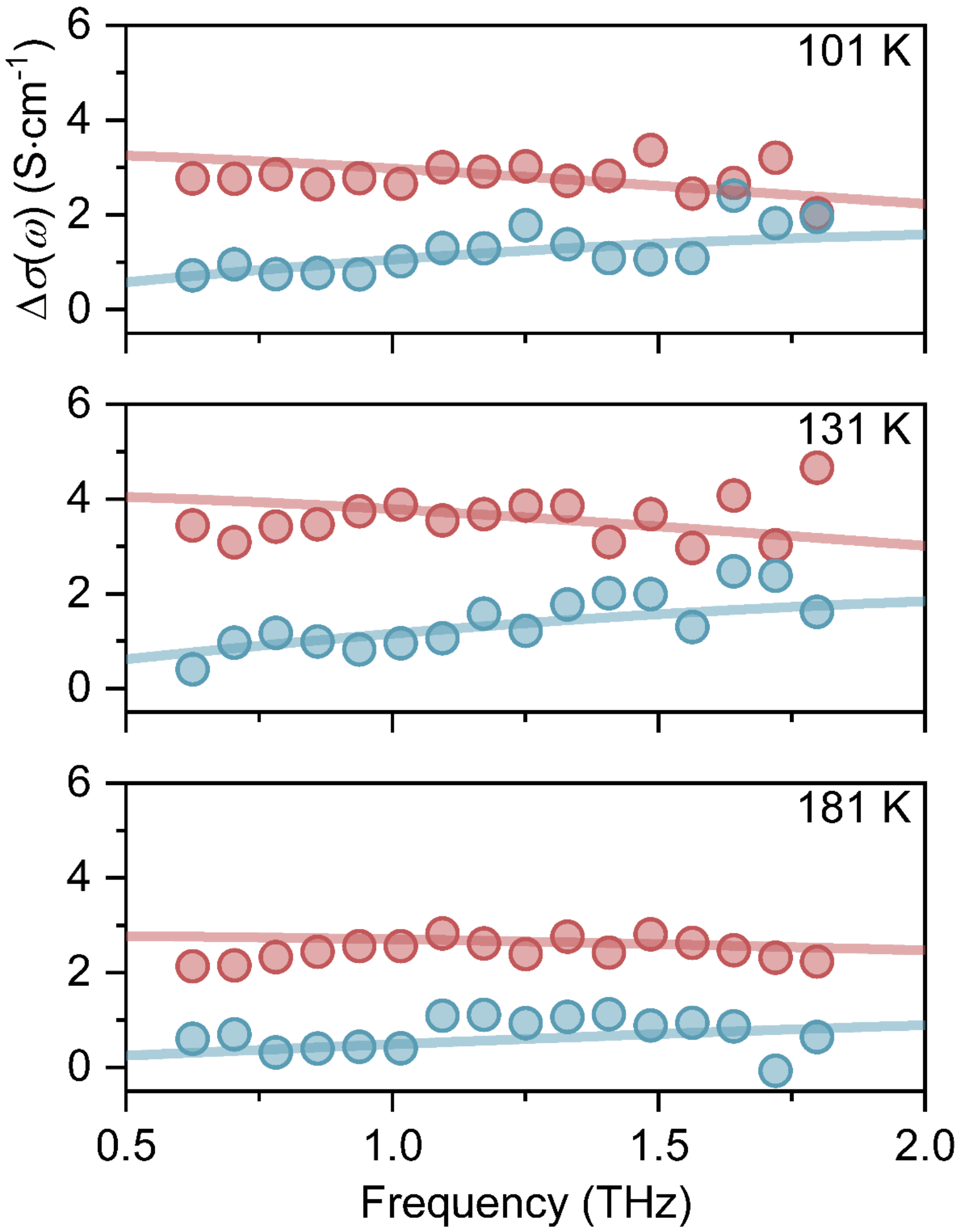

Supplementary Fig. 16 Frequency-resolved complex THz photoconductivity, $\Delta\sigma(\omega)$, measured at 2 ps after σ_{peak} for PI-DY2DP at 101 K (top), 131 K (middle), and 181 K (bottom). Red and blue symbols denote the real and imaginary components of $\Delta\sigma(\omega)$, respectively, and solid lines represent the corresponding Drude fits.

Supplementary Table 1 DFT calculated carrier masses of PI-DY2DP and PF-DY2DP.

	PI-DY2DP (m_0)	PF-DY2DP (m_0)
Interlayer hole mass (Γ–N)	0.24	>10
Interlayer electron mass (Γ–N)	0.74	>10
Intralayer hole mass (X–M–Y)	0.15	0.12
Intralayer electron mass (X–M–Y)	0.33	0.38

Supplementary Table 2 A comparison of charge mobilities of 2DPs and 2D COFs.

Sample name	μ ($cm^2\ V^{-1}\ s^{-1}$)	References
PI-DY2DP	480 ± 50[a]	This work
1D-Pery-COF	66 ± 14[b]	[2]
2D ML- Pery-COF	49 ± 10[b]	[2]
2D PL-Pery-COF	21 ± 4[b]	[2]
CuPc-MIDA-COF	13.3 ± 7.5[b]	[3]
CuPc-MIDA-COF	8.3[c]	[3]
HHTP-MIDA-COF	3.4 ± 2.5[b]	[3]
H_2P-COF	3.5[d]	[4]
CuP-COF	0.19[d]	[4]
ZnP-COF	0.048[d]	[4]
NiPc-COF	1.3[d]	[5]
NiPc-BTDA COF	0.6[d]	[6]
COF-366	8.1[d]	[7]
COF-66	3[d]	[7]
2D D-A COF	0.05[d]	[8]
CS-COF	4.2[d]	[9]
TTF-Ph-COF	0.2[d]	[10]
TTF-Py-COF	0.08[d]	[10]
HBC-COF (film)	0.7[d]	[11]
CuPc-pz COF	0.9 ± 0.2[c]	[12]
ZnPc-pz COF	4.8 ± 0.7[c]	[12]
NiPc-CoTAA (film)	0.15[c]	[13]
BUCT-COF-1	2.75 ± 0.22[c]	[14]
DBOV-COF (film)	0.6 ± 0.1[a]	[15]
sp^2c-COF	22.1 ± 2.7[a]	[16]
sp^2c-COF-6	2.3 ± 0.5[a]	[16]
sp^2c-COF-8	< 0.1[a]	[16]
sp^2c-COF-9	5.8 ± 0.9[a]	[16]
c-HBC-COF	44[a]	[17]
TPB-TFB COF (film)	165 ± 10[a]	[18]
V-2D-COF-W1	1.4[a]	[19]
V-2D-COF-W3	10.3[a]	[19]

V-2D-COF-W4	0.6[a]	19
2DPAV-BDT-BT	65[a]	20
2DPAV-BDT-BP	17[a]	20
2DCP-NiPc (film)	971 ± 44[a]	21
2DCP-CuPc (film)	460 ± 31[a]	21
AntTTH	~0.1[d]	22
Joa-COF-2	14 ± 1[a]	23
2DPI-BTT-BP	~1[a]	24
2DPAV-BTT-BP	~13[a]	24
2DPAV-TBDT-DPP-1	170 ± 40[a]	25
2DPAV-TBDT-DPP-1	310 ± 50[a]	25

[a]Mobility obtained by time-resolved THz spectroscopy taking into account the backscattering effect; [b]Mobility obtained by time-resolved THz spectroscopy without taking into account the backscattering effect; [c]Mobility obtained by Hall effect measurements; [d]Mobility obtained by flash-photolysis time-resolved microwave conductivity.

Supplementary Table 3 A comparison of photon-to-free-carrier conversion ratios of 2DPs and 2D COFs.

Sample name	η	References
PI-DY2DP	42%[a]	This work
COF-66	1.5×10^{-5}, [b]	[7]
COF-366	1.7×10^{-5}, [b]	[7]
HBC-COF	2.1×10^{-5}, [b]	[11]

[a]Photon-to-free-carrier conversion ratio estimated by TRTS; [b]Photon-to-free-carrier conversion ratio estimated by combining flash photolysis time-resolved microwave conductivity, combined with the current integration method.

References

1 Fu, S. *et al.* Unveiling high-mobility hot carriers in a two-dimensional conjugated coordination polymer. *Nat. Mater.* **24**, 1457-1464 (2025).

2 Fu, S. *et al.* Dimensional evolution of charge mobility and porosity in covalent organic frameworks. *Nat. Commun.* **16**, 2219 (2025).

3 Jin, E. *et al.* Exceptional electron conduction in two-dimensional covalent organic frameworks. *Chem* **7**, 3309-3324 (2021).

4 Feng, X. *et al.* High-rate charge-carrier transport in porphyrin covalent organic frameworks: Switching from hole to electron to ambipolar conduction. *Angew. Chem. Int. Ed.* **51**, 2618-2622 (2012).

5 Chumakov, Y., Aksakal, F., Dimoglo, A., Ata, A. & Palomares-Sánchez, S. A. First-principles study of thermoelectric properties of covalent organic frameworks. *J. Electron. Mater.* **45**, 3445-3452 (2016).

6 Ding, X. *et al.* An n-channel two-dimensional covalent organic framework. *J. Am. Chem. Soc.* **133**, 14510-14513 (2011).

7 Wan, S. *et al.* Covalent organic frameworks with high charge carrier mobility. *Chem. Mater.* **23**, 4094-4097 (2011).

8 Feng, X. *et al.* An ambipolar conducting covalent organic framework with self-sorted and periodic electron donor-acceptor ordering. *Adv. Mater.* **24**, 3026-3031 (2012).

9 Guo, J. *et al.* Conjugated organic framework with three-dimensionally ordered stable structure and delocalized π clouds. *Nat. Commun.* **4**, 2736 (2013).

10 Jin, S. *et al.* Two-dimensional tetrathiafulvalene covalent organic frameworks: Towards latticed conductive organic salts. *Chem. Eur. J.* **20**, 14608-14613 (2014).

11 Dalapati, S. *et al.* Rational design of crystalline supermicroporous covalent organic frameworks with triangular topologies. *Nat. Commun.* **6**, 7786 (2015).

12 Wang, M. *et al.* Unveiling electronic properties in metal–phthalocyanine-based pyrazine-linked conjugated two-dimensional covalent organic frameworks. *J. Am. Chem. Soc.* **141**, 16810-16816 (2019).

13 Yue, Y. *et al.* Conductive metallophthalocyanine framework films with high carrier mobility as efficient chemiresistors. *Angew. Chem. Int. Ed.* **60**, 10806-10813 (2021).

14 Wang, S. *et al.* A fully conjugated 3D covalent organic framework exhibiting band-like transport with ultrahigh electron mobility. *Angew. Chem. Int. Ed.* **60**, 9321-9325 (2021).

15 Jin, E. *et al.* A nanographene-based two-dimensional covalent organic framework as a stable and efficient photocatalyst. *Angew. Chem. Int. Ed.* **61**, e202114059 (2022).

16 Jin, E. *et al.* Module-patterned polymerization towards crystalline 2D sp^2-carbon covalent organic framework semiconductors. *Angew. Chem. Int. Ed.* **61**, e202115020 (2022).

17 Xing, G. *et al.* Nonplanar rhombus and kagome 2D covalent organic frameworks from distorted aromatics for electrical conduction. *J. Am. Chem. Soc.* **144**, 5042-5050 (2022).

18 Fu, S. *et al.* Outstanding charge mobility by band transport in two-dimensional semiconducting covalent organic frameworks. *J. Am. Chem. Soc.* **144**, 7489-7496 (2022).

19 Liu, Y. *et al.* Vinylene-linked 2D conjugated covalent organic frameworks by Wittig reactions. *Angew. Chem. Int. Ed.* **61**, e202209762 (2022).

20 Liu, Y. *et al.* A thiophene backbone enables two-dimensional poly(arylene vinylene)s with high charge carrier mobility. *Angew. Chem. Int. Ed.* **62**, e202305978 (2023).

21 Wang, M. *et al.* Exceptionally high charge mobility in phthalocyanine-based poly(benzimidazobenzophenanthroline)-ladder-type two-dimensional conjugated polymers. *Nat. Mater.* **22**, 880-887 (2023).

22 Ghosh, S. *et al.* Low band gap semiconducting covalent organic framework films with enhanced photocatalytic hydrogen evolution. *J. Mater. Chem. A* **12**, 247-255 (2024).

23 Almarza, J. *et al.* High charge carrier mobility in non-conjugated 3D covalent organic frameworks. *Adv. Mater.* e17211 (2026).

24 Ghouse, S. *et al.* Towards single-crystalline two-dimensional poly(arylene vinylene) covalent organic frameworks. *Nat. Chem.* doi:10.1038/s41557-025-02048-8 (2026).

25 Zhao, R. *et al.* Diketopyrrolopyrrole-based two-dimensional poly(arylene vinylene)s with high charge carrier mobility. *Nat. Commun.* **17**, 1348 (2026).
