# Supplementary material for: Suppressed excitonic effects enable high mobility and high-yield photoconductivity in a pyridine-coordinated two-dimensional polymer crystal
Source: Nat Commun. 2026 Aug 21;17:8815. doi: 10.1038/s41467-026-77017-x (PMC13498572; doi:10.1038/s41467-026-77017-x)
Supplement: Supplementary file 2 [file 41467_2026_77017_MOESM2_ESM.pdf]

nature portfolio

Peer Review File

Suppressed excitonic effects enable high mobility and high-yield photoconductivity in a pyridine-coordinated two-dimensional polymer crystal

Corresponding Author: Professor Xinliang Feng

This file contains all reviewer reports in order by version, followed by all author rebuttals in order by version.

Version 0:

Reviewer comments:

Reviewer #1

(Remarks to the Author)
In the manuscript, Fu et al. leverage axial pyridine coordination to suppress excitonic effects in a diyne-linked two-dimensional polymer (2DP) crystal synthesized on the surface of a dimethylacetamide-water mixture, enabling inorganic-like charge transport properties, including a high charge carrier mobility of 500 $cm^2·V^{-1}·s^{-1}$ and a photon-to-free-carrier conversion ratio of 40%. The combination of rational interlayer coordination design, on-liquid surface synthesis, ultrafast spectroscopic investigation, and theoretical calculations establishes a coherent framework for developing new 2DPs with exceptional optoelectronic properties, well aligned with the interdisciplinary scope of the journal. The proposed design strategy, along with the fundamental insights into charge transport and recombination mechanisms, is valuable and will likely guide further efforts in structural design, defect engineering, and optoelectronic device integration in this field. Overall, the discussions and conclusions are well supported by the data, and the manuscript is clearly written. I therefore recommend publication in Nature Communications following revisions and clarifications:
The manuscript lacks a quantitative statistical assessment of crystal sizes, which is critical for evaluating their potential impact on charge transport properties.
The XRS characterization should be further analyzed with particular attention to the carbon–carbon triple bonds. A more detailed spectral assignment and discussion are necessary to confirm the presence, chemical environment, and possible electronic contribution of the C≡C units.
The crystals in the OM image (Figure 1c) appear heterogeneous. Could the authors comment on the possible origin of this heterogeneity? In addition, the authors should clarify whether such heterogeneity affects optical measurements.
In Figure 4e, the authors compare the dynamic features obtained from transient absorption spectroscopy and time-resolved terahertz spectroscopy to elucidate the photoconductivity decay mechanism. While the temporal overlap supports the conclusion that “the initial drop in photoconductivity originates predominantly from ultrafast non-radiative free-carrier recombination, rather than conversion into long-lived excitons”, additional clarification regarding data processing is needed. Specifically, the authors should clarify: (1) whether the transient absorption signal corresponds to a single selected wavelength or an integration over a photoinduced absorption band; (2) the pump wavelength and excitation density used for the photoconductivity measurements.
The fast decay of photoconductivity and carrier population is attributed to the presence of shallow trap states. While it is understood that identifying the precise nature and density of such trap states is challenging, the authors are encouraged to include a brief discussion on how trap states may influence band structure and charge transport in this class of materials, supported by prior experimental or theoretical studies.
Figure 3b presents frequency-resolved complex THz photoconductivity at different time delays. It is unclear whether these time delays correspond to those shown in Figures 3c and 3d. The authors should clarify this point and explicitly label the specific time delay associated with each curve in Figure 3b to improve clarity.

Reviewer #2

(Remarks to the Author)
This is an incredibly well-designed, highly orchestrated collaboration between synthetic, structure characterization and measurement focused groups to investigate a new 3D organic-based conductive system. The report of this new material

design deserves rapid publication since many research groups working to generate highly mobile materials other than conventional solid-state semiconductors will be highly interested and inspired by these findings. I applaud the authors for their concerted efforts and strongly urge Nature Comm to accept this submission without delay.

The paper is extremely well-written and easily read and clear. All findings are explained, evidence supported and compared to earlier related studies leading to future related studies, reproduction by other groups, and fully comprehended by all readers.

Perhaps the authors will consider:

1) The most important finding, as proposed and found for the 3D design, is high mobility without issues found with earlier conducting polymer film systems (by THz methods) - localized exciton recombination and reduced mobility due to phase segregation, etc. A mobility value of ca. 500 $cm2·V^{-1}·s^{-1}$ is enormous! While conductivity values of ca. 10 S/cm is not unusual for highly conductive polymers (e.g., IDTPT, PSBTBT) the new 3D COF design with pyridine linkage attains high mobility because the number density of photon-to-carrier conversion efficiency is proposed to be large. Perhaps the authors could show this (rather than comparing to published results) by a direct, under same experimental conditions comparison with a 2D "standard" polymer film (as in Fig. 2f and 2g)?

2) Communications paper restrictions make it difficult to show and explain many items at once. It appears the authors have managed this extremely well, but Figures 1 and 2 that include many subjects appears (to this reviewer) as an attempt to condense a longer more detailed article. Could many of the sub-figures be simply referenced in the SI (without duplication)? For example, Fig. 3 is easily understood and to the point but Fig. 3b is lacking y-axis values for missing delay times.

Conclusions: Can this material be incorporated into a solar cell and compared to Si (with efficiencies of ca. 25%) and above the Shottky-Quisser ca. 35% limit? If so, that would be incredible!

I hope the authors continue to develop new and exciting materials and investigative methods to advance the field of photon-to-electron conversion and thank them for generating this exciting addition to the literature.

Version 1:

Reviewer comments:

Reviewer #1

(Remarks to the Author)
The revision details can be provided and so it can be suggested for accepatance.

Reviewer #2

(Remarks to the Author)
Dear Authors,

Thank you so much for your consideration of both reviewers' comments and suggestions concerning your highly important and pertinent study and findings.

I thank you for taking time and responding to all reviewer comments and making revisions to your manuscript that expertly addressed all issues!

Looking forward to seeing your contribution in print very soon and good luck with all next steps towards incorporating your novel material into potentially highly efficient solar cells.

Reviewer 2

Open Access This Peer Review File is licensed under a Creative Commons Attribution 4.0 International License, which permits use, sharing, adaptation, distribution and reproduction in any medium or format, as long as you give appropriate credit to the original author(s) and the source, provide a link to the Creative Commons license, and indicate if changes were made.
In cases where reviewers are anonymous, credit should be given to 'Anonymous Referee' and the source.
The images or other third party material in this Peer Review File are included in the article's Creative Commons license, unless indicated otherwise in a credit line to the material. If material is not included in the article's Creative Commons license and your intended use is not permitted by statutory regulation or exceeds the permitted use, you will need to obtain permission directly from the copyright holder.
To view a copy of this license, visit https://creativecommons.org/licenses/by/4.0/

Reviewer #1 (Remarks to the Author):

In the manuscript, Fu et al. leverage axial pyridine coordination to suppress excitonic effects in a diyne-linked two-dimensional polymer (2DP) crystal synthesized on the surface of a dimethylacetamide-water mixture, enabling inorganic-like charge transport properties, including a high charge carrier mobility of 500 $cm^2 \cdot V^{-1} \cdot s^{-1}$ and a photon-to-free-carrier conversion ratio of 40%. The combination of rational interlayer coordination design, on-liquid surface synthesis, ultrafast spectroscopic investigation, and theoretical calculations establishes a coherent framework for developing new 2DPs with exceptional optoelectronic properties, well aligned with the interdisciplinary scope of the journal. The proposed design strategy, along with the fundamental insights into charge transport and recombination mechanisms, is valuable and will likely guide further efforts in structural design, defect engineering, and optoelectronic device integration in this field. Overall, the discussions and conclusions are well supported by the data, and the manuscript is clearly written. I therefore recommend publication in Nature Communications following revisions and clarifications:

Response: We greatly appreciate the reviewer's recognition of the significance of our design strategy and the overall quality of the manuscript. Below, we provide a detailed, point-by-point response to the remaining comments and suggestions.

Comment 1: The manuscript lacks a quantitative statistical assessment of crystal sizes, which is critical for evaluating their potential impact on charge transport properties.

Response: We thank the reviewer for this comment. In response, we have carried out a quantitative statistical analysis of the crystal dimensions. As shown in Fig. R1, the crystals exhibit a unimodal size distribution, with widths ranging from 0.3 to 0.6 μm and lengths spanning 6 to 14 μm. These results confirm the formation of rod-shaped PI-DY2DP crystals with characteristic micrometer-scale dimensions.

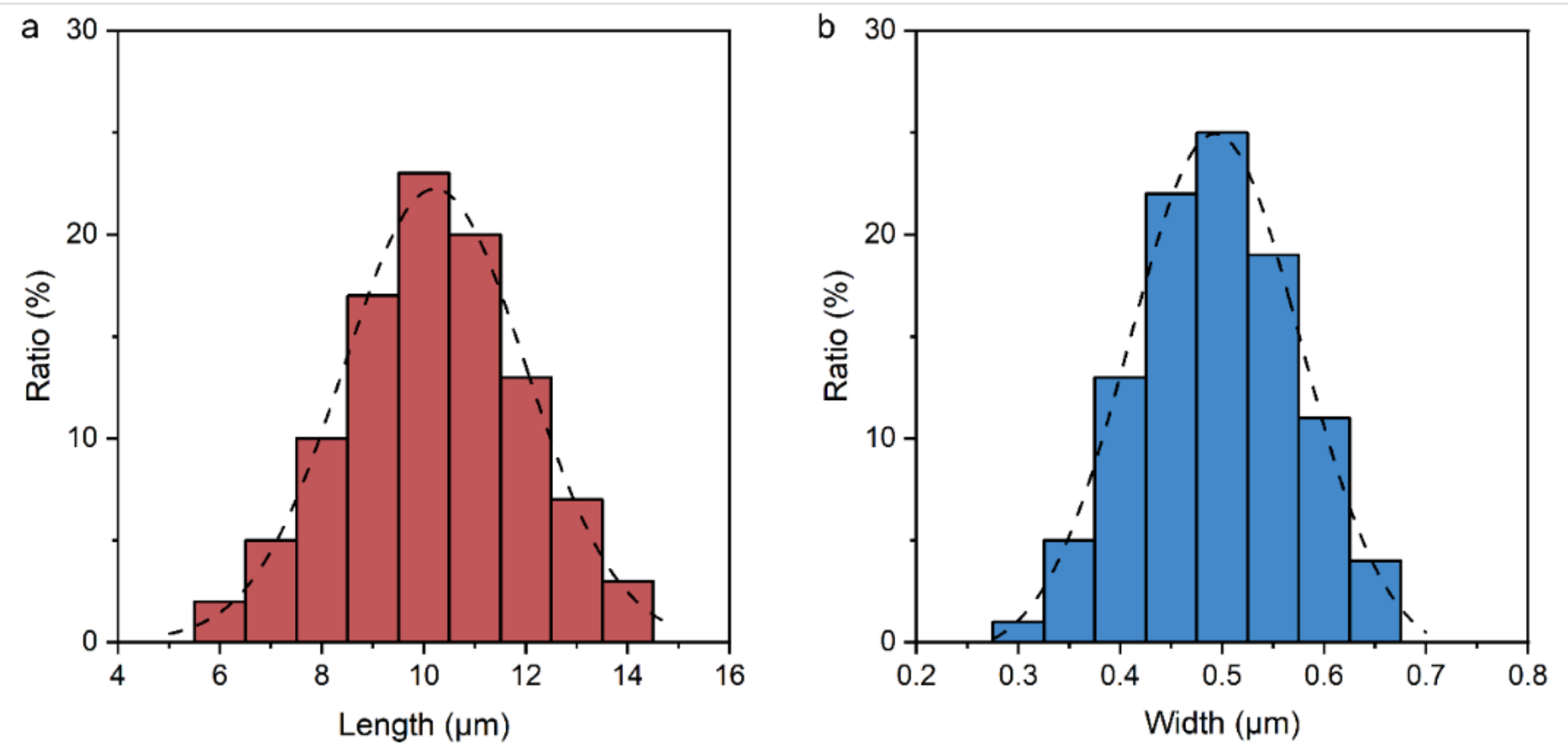

Fig. R1. Statistical analysis of PI-DY2DP crystal dimensions. **a**, Length distribution. **b**, Width distribution.

Actions:

(i) We have added the following description to page 7 of the revised manuscript: "...Polarized optical microscopy (OM) and corresponding statistical analysis of PI-DY2DP crystal dimensions reveal the formation of rod-shaped crystals and a unimodal size distribution, exhibiting widths of 0.3-0.6 μm and lengths of 6-14 μm (**Fig. 1c** and Supplementary Fig. 3)...."

(ii) Fig. R1 has been included as Supplementary Fig. 3 in the Supplementary Information.

Comment 2: The XRS characterization should be further analyzed with particular attention to the carbon–carbon triple bonds. A more detailed spectral assignment and discussion are necessary to confirm the presence, chemical environment, and possible electronic contribution of the C≡C units.

Response: Following the reviewer's suggestion, we have performed a detailed deconvolution and assignment of the C *1s* XPS spectrum, with particular emphasis on the C≡C units. As shown in Fig. R2, in addition to the primary components assigned to C-C/C=C (~284.3 eV) and C=N (~285.8 eV), a distinct feature at ~285.1 eV is resolved. This peak is attributed to sp-hybridized carbon atoms associated with C≡C bonds. This assignment is supported by the higher s-character of sp-hybridized orbitals, which leads to increased electron density closer to the nucleus and thus a slightly higher binding energy compared to sp^2-hybridized carbon species. The observed spectral features are therefore fully consistent with the expected chemical structure of the PI-DY2DP framework and confirm the successful formation of diyne linkages.

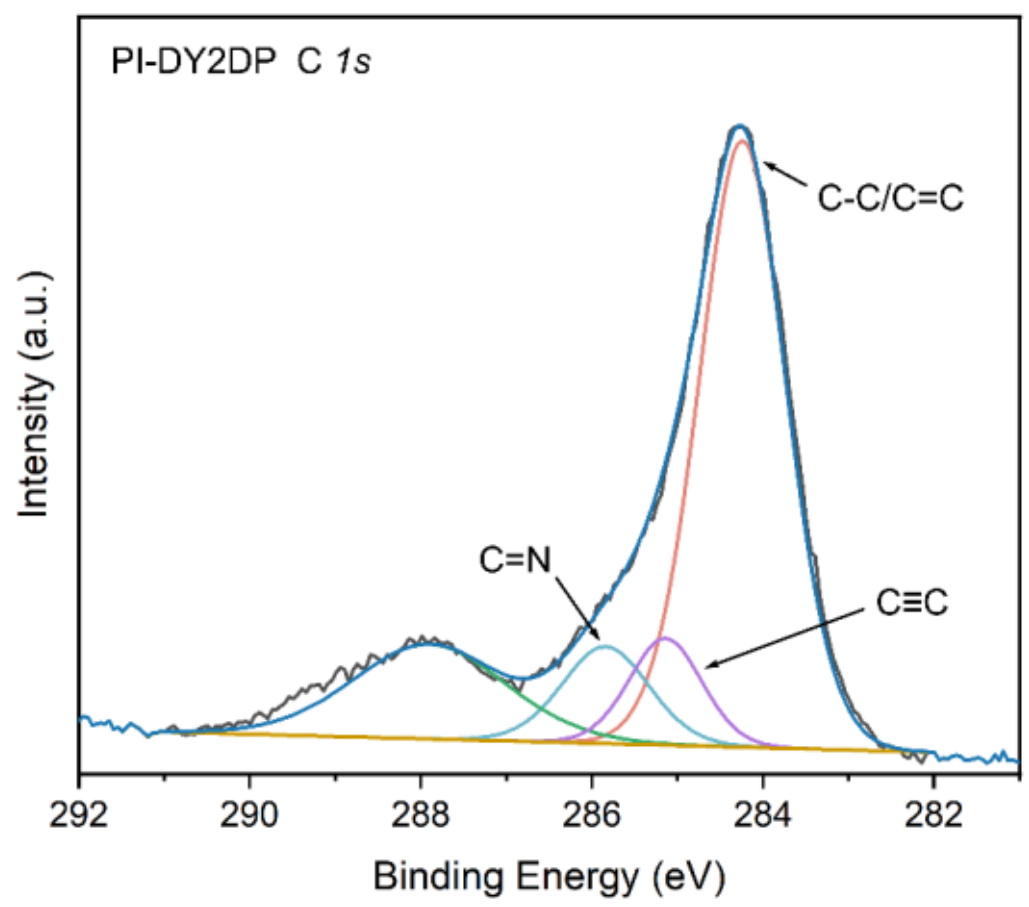

Fig. R2. High-resolution XPS C *1s* spectrum of PI-DY2DP.

Actions:

(i) We have added the following description to page 7 of the revised manuscript: "... Complementary analysis of the C *1s* region reveals, in addition to the dominant C–C/C=C (~284.3 eV) and C=N (~285.8 eV) components, a distinct feature at ~285.1 eV can be assigned to sp-hybridized carbon atoms associated with C≡C bonds (Supplementary Fig. 6). This assignment is consistent with the higher s-character of sp-hybridized orbitals, which results in a slight positive shift in binding energy relative to sp^2 carbon species, thereby confirming the formation of diyne linkages in the framework....";

(ii) Fig. R2 has been included as Supplementary Fig. 6 in the Supplementary Information.

Comment 3: The crystals in the OM image (Figure 1c) appear heterogeneous. Could the authors comment on the possible origin of this heterogeneity? In addition, the authors should clarify whether such heterogeneity affects optical measurements.

Response: We thank the reviewer for pointing this out. The slight heterogeneity observed in the OM image likely originates from subtle interfacial perturbations during crystal growth, which can influence monomer alignment on the liquid surface. Given that precise alignment is critical for achieving extended conjugation and high crystallinity in PI-DY2DP, even minor deviations can propagate during growth, resulting in crystals with marginally different orientations and, consequently, the observed heterogeneity in the OM image. Nevertheless, this heterogeneity does not affect the optical measurements. As demonstrated by the reproducibility tests (previously shown in Supplementary Fig. 10), both the photoconductivity amplitude and carrier dynamics

remain highly consistent across different sample regions, confirming the robustness and reliability of the measured optoelectronic properties.

Action:

We have added the following discussion to page 7 in the revised manuscript: "...The slight heterogeneity observed in the OM image is likely due to subtle interfacial perturbations that arise during crystal growth. Such perturbations can affect the alignment of the monomers at the interface; even minor deviations may propagate during growth, leading to marginal orientational variations...."

Comment 4: In Figure 4e, the authors compare the dynamic features obtained from transient absorption spectroscopy and time-resolved terahertz spectroscopy to elucidate the photoconductivity decay mechanism. While the temporal overlap supports the conclusion that "the initial drop in photoconductivity originates predominantly from ultrafast non-radiative free-carrier recombination, rather than conversion into long-lived excitons", additional clarification regarding data processing is needed. Specifically, the authors should clarify: (1) whether the transient absorption signal corresponds to a single selected wavelength or an integration over a photoinduced absorption band; (2) the pump wavelength and excitation density used for the photoconductivity measurements.

Response: We agree with the reviewer that clarifying the data processing and experimental conditions can improve the transparency of the comparison. Specifically, the transient absorption signal is obtained by integrating the high-energy PIB band between 410 and 470 nm. The photoconductivity measurements were performed using a pump wavelength of 400 nm with an N_{abs} of 7.00×10^{12} cm^{-2}. These details have now been clarified in the revised manuscript.

Action:

We have added the following information to the caption of Fig. 4e in the revised manuscript: "**e,** Comparison of normalized carrier dynamics extracted from TRTS (red line, 3.10 eV photoexcitation at N_{abs} = in 7.00×10^{12} cm^{-2}) and TA (blue dots, 3.10 eV photoexcitation at N_{abs} = 2.9×10^{12} cm^{-2}, integrated over the high-energy PIB band between 410 and 470 nm shown in **d**)."

Comment 5: The fast decay of photoconductivity and carrier population is attributed to the presence of shallow trap states. While it is understood that identifying the precise nature and

density of such trap states is challenging, the authors are encouraged to include a brief discussion on how trap states may influence band structure and charge transport in this class of materials, supported by prior experimental or theoretical studies.

Response: Following this comment, we have added the following discussion to page 14 of the revised manuscript on the potential roles of trap states in modulating the band structure and charge transport in organic two-dimensional crystals:

"...Previous experimental and theoretical studies have shown that defects can profoundly influence charge transport in O2DCs through multiple mechanisms, including acting as recombination centers, modulating carrier density, altering the electronic band structure (e.g., bandgap and band dispersion), introducing charge scattering sites, and affecting the local phonon density of states and inter-grain hopping barriers[11]...."

Comment 6: Figure 3b presents frequency-resolved complex THz photoconductivity at different time delays. It is unclear whether these time delays correspond to those shown in Figures 3c and 3d. The authors should clarify this point and explicitly label the specific time delay associated with each curve in Figure 3b to improve clarity.

Response: In response, and in line with a similar comment from Reviewer 2, we have revised Figure 3b to improve clarity. Specifically, we have simplified the presentation and explicitly labeled the time delay corresponding to each curve in the figure. The revised figure is provided below.

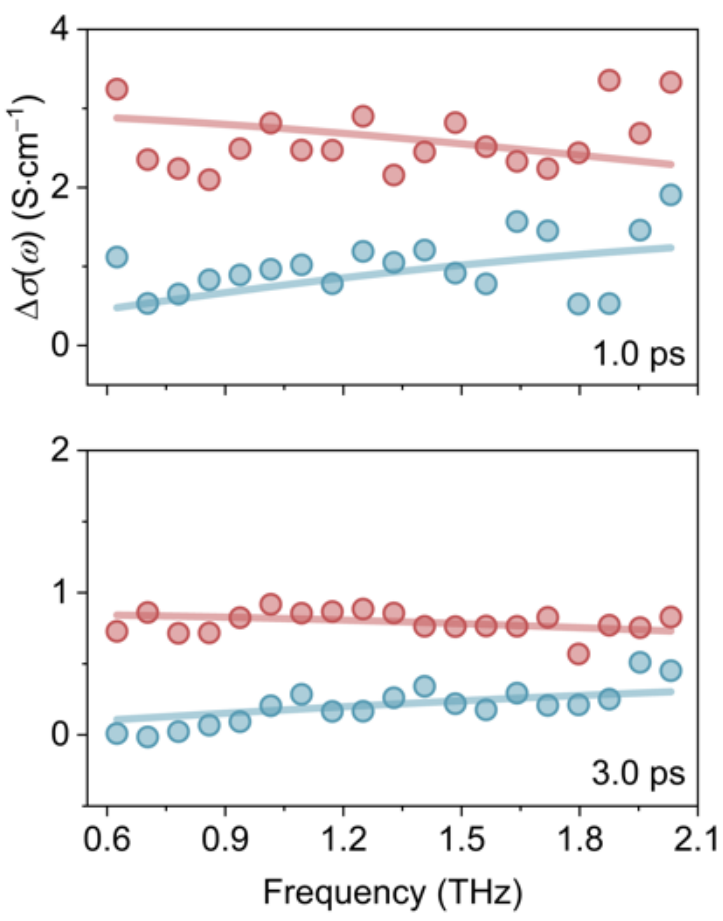

Fig. 3b. Frequency-resolved complex THz photoconductivity, $\Delta\sigma(\omega)$, at 1 ps (top) and 3 ps (bottom) after $\Delta\sigma_{\text{peak}}$. Red and blue symbols denote the real and imaginary components of $\Delta\sigma(\omega)$, respectively, and solid lines show the corresponding Drude fits.

Reviewer #2 (Remarks to the Author):

This is an incredibly well-designed, highly orchestrated collaboration between synthetic, structure characterization and measurement focused groups to investigate a new 3D organic-based conductive system. The report of this new material design deserves rapid publication since many research groups working to generate highly mobile materials other than conventional solid-state semiconductors will be highly interested and inspired by these findings. I applaud the authors for their concerted efforts and strongly urge Nature Comm to accept this submission without delay. The paper is extremely well-written and easily read and clear. All findings are explained, evidence supported and compared to earlier related studies leading to future related studies, reproduction by other groups, and fully comprehended by all readers.

Response: We sincerely thank the reviewer for the highly positive and encouraging evaluation of our work.

Comment 1: Perhaps the authors will consider: 1) The most important finding, as proposed and found for the 3D design, is high mobility without issues found with earlier conducting polymer film systems (by THz methods) - localized exciton recombination and reduced mobility due to phase segregation, etc. A mobility value of ca. 500 $cm^2 \cdot V^{-1} \cdot s^{-1}$ is enormous! While conductivity values of ca. 10 S/cm is not unusual for highly conductive polymers (e.g., IDTPT, PSBTBT) the new 3D COF design with pyridine linkage attains high mobility because the number density of photon-to-carrier conversion efficiency is proposed to be large. Perhaps the authors could show this (rather than comparing to published results) by a direct, under same experimental conditions comparison with a 2D "standard" polymer film (as in Fig. 2f and 2g)?

Response: We thank the reviewer for this insightful suggestion. To provide a meaningful benchmark, we have included a comparison with Cu_3BHT (BHT = benzenehexathiol), a well-established electrically conductive 2D coordination polymer with excellent charge transport properties (*Nat. Commun.*, **2015**, 6, 7408). Notably, although Cu_3BHT exhibits highly mobile hot carriers immediately after 3.10 eV excitation (*Nat. Mater.*, **2025**, 24, 1457-1464), it requires an absorbed photon density more than two orders of magnitude higher to achieve a photoconductivity comparable to that of PI-DY2DP. This comparison highlights the markedly enhanced photon-to-free-carrier conversion efficiency in PI-DY2DP.

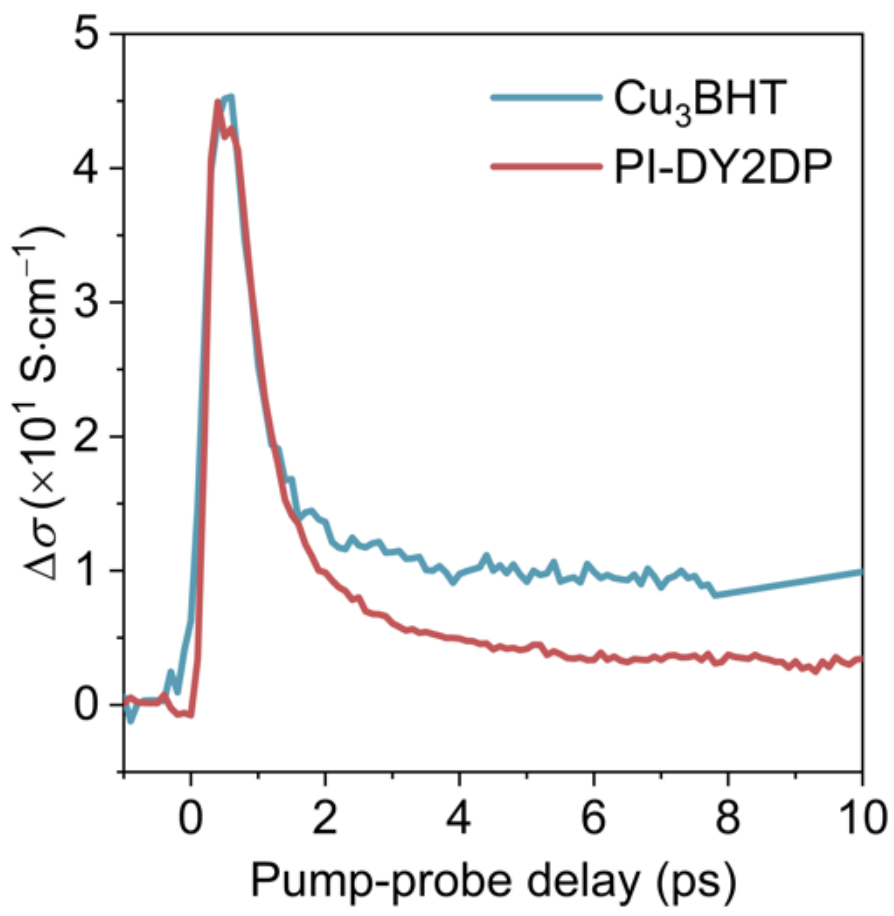

Fig. R1. Comparison of time-resolved $\Delta\sigma$ of Cu_3BHT (BHT = benzenehexathiol) (3.10 eV photoexcitation at $N_{abs} = 9.60 \times 10^{14}$ cm^{-2}) and PI-DY2DP (3.10 eV photoexcitation at $N_{abs} = 7.00 \times 10^{12}$ cm^{-2}, same as in Fig. 2g) at room temperature. Data for Cu_3BHT are reproduced from *Nat. Mater.*, **2025**, 24, 1457-1464.

Action:

(i) We have added the following discussion to page 11 of the revised manuscript: "...To further benchmark performance, we compared PI-DY2DP with Cu_3BHT (BHT = benzenehexathiol), a well-established electrically conductive 2D coordination polymer[43]. Following 3.10 eV photoexcitation, Cu_3BHT requires an N_{abs} more than two orders of magnitude higher to achieve a $\Delta\sigma$ comparable to that of PI-DY2DP, despite exhibiting highly mobile hot carriers at the photoconductivity maximum (Supplementary Fig. 13)[44]. This comparison highlights the markedly enhanced ϕ in PI-DY2DP....";

(ii) Fig. R1 has been included as Supplementary Fig. 13 in the Supplementary Information.

Comment 2: 2) Communications paper restrictions make it difficult to show and explain many items at once. It appears the authors have managed this extremely well, but Figures 1 and 2 that include many subjects appears (to this reviewer) as an attempt to condense a longer more detailed article. Could many of the sub-figures be simply referenced in the SI (without duplication)? For example, Fig. 3 is easily understood and to the point but Fig. 3b is lacking y-axis values for missing delay times.

Response: We thank the reviewer for this helpful suggestion. To improve clarity and readability, we have (i) simplified Figure 3b by moving several subpanels to Supplementary Fig. 15 in the

Supplementary Information, and (ii) added the previously missing y-axis values and explicitly labelled the corresponding delay times for each dataset. The revised figure is provided below.

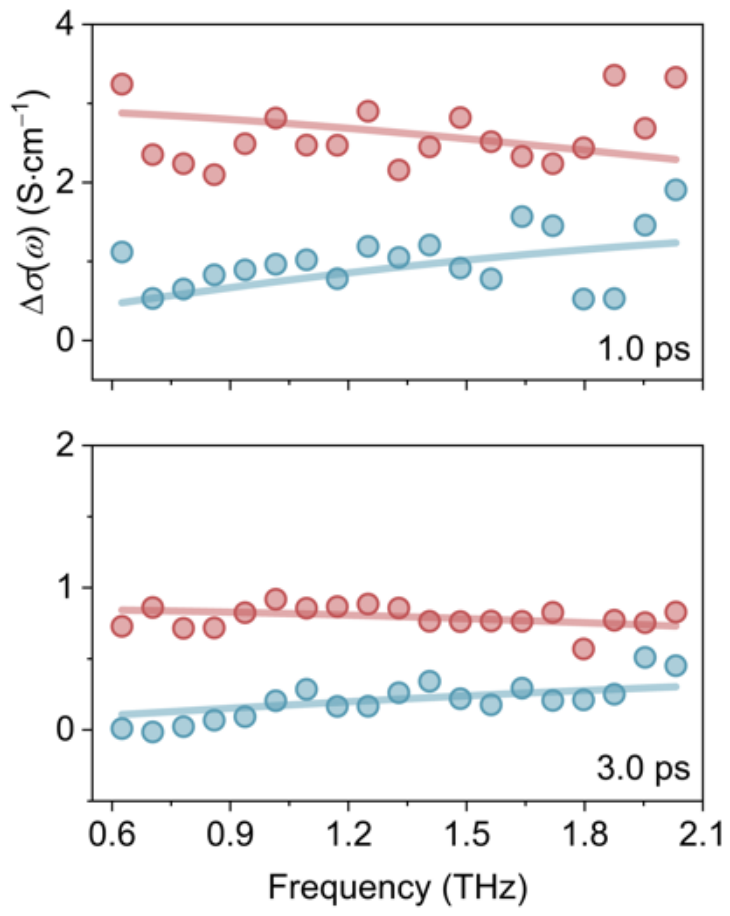

Fig. 3b. Frequency-resolved complex THz photoconductivity, $\Delta\sigma(\omega)$, at 1 ps (top) and 3 ps (bottom) after $\Delta\sigma_{\text{peak}}$. Red and blue symbols denote the real and imaginary components of $\Delta\sigma(\omega)$, respectively, and solid lines show the corresponding Drude fits.

Comment 3: Conclusions: Can this material be incorporated into a solar cell and compared to Si (with efficiencies of ca. 25%) and above the Shottky-Quisser ca. 35% limit? If so, that would be incredible! I hope the authors continue to develop new and exciting materials and investigative methods to advance the field of photon-to-electron conversion and thank them for generating this exciting addition to the literature.

Response: We thank the reviewer for this forward-looking and encouraging comment. We fully agree that exploring the photovoltaic potential of this material is an exciting direction, which is closely aligned with our ongoing research efforts. At the current stage, however, meaningful device-level benchmarking is not yet feasible, as several key challenges remain. In particular, further advances are required in (i) defect engineering to extend carrier lifetimes, (ii) optimization of synthesis protocols to achieve large-area thin films suitable for device integration, and (iii) identification of appropriate contact materials to enable efficient charge extraction. Motivated by the reviewer's suggestion, we are actively pursuing these directions. We anticipate that addressing these challenges will be critical for the successful integration of this material into photovoltaic architectures and, ultimately, for achieving competitive photon-to-electricity conversion efficiencies. We look forward to reporting progress along these lines in future studies.
